# Lung Manifestations of Metastatic Duodenal Adenocarcinoma: A Case Report

**DOI:** 10.7759/cureus.91537

**Published:** 2025-09-03

**Authors:** Asmitha P Reddy, Zeyad J Rifai, Mingchen Song, Mohamad Bahrou, Saif Faiek

**Affiliations:** 1 Internal Medicine, Southern Illinois University School of Medicine, Springfield, USA; 2 Pulmonary and Critical Care Medicine, Southern Illinois University School of Medicine, Springfield, USA

**Keywords:** duodenal adenocarcinoma, lymphatic spread, metastasis, pulmonary lymphangitic carcinomatosis, pulmonary metastasis

## Abstract

Pulmonary lymphangitic carcinomatosis (PLC) is a rare condition characterized by malignant infiltration within the lymphatic system. It most often arises from adenocarcinoma of the breast, lung, or pancreas. This rare diagnosis often evades detection due to diagnostic challenges, unknown pathogenesis, and indolent presentation. Villous adenoma of the duodenum is also an exceedingly rare malignancy that is often mistaken for various pancreatic pathologies. To our knowledge, we describe the first case of PLC secondary to biopsy-confirmed villous adenocarcinoma of the duodenum in a 56-year-old male who presented with dyspnea, cough, and weight loss. This case illustrates how an indolent-appearing duodenal lesion can manifest as aggressive pulmonary metastasis, emphasizing the need for clinicians to maintain a broad differential when evaluating diffuse pulmonary disease and the importance of integrating radiographic, histopathologic, molecular, and multidisciplinary tools to identify rare metastatic origins.

## Introduction

Pulmonary lymphangitic carcinomatosis (PLC) is a rare condition characterized by malignant infiltration of the pulmonary lymphatic system. It constitutes about 6% of metastatic pulmonary malignancies, originating predominantly from primary adenocarcinomas, with breast, lung, and gastric cancers accounting for two-thirds of the cases, while less common origins, such as the pancreas, colon, thyroid, cervix, and prostate, are reported rarely [1]. Dyspnea, nonproductive cough, and weight loss are the typical presenting symptoms, as with most pulmonary malignancies [1]. Unfortunately, diagnosis often occurs postmortem due to its rarity; therefore, the true incidence is unknown.

Duodenal polyps are an uncommon finding upon endoscopic evaluation and are reported in 0.3%-4.6% of individuals who undergo upper endoscopy [2]. Among these, non-ampullary duodenal adenomas arise sporadically in patients without hereditary polyposis syndromes, such as familial adenomatous polyposis or Gardner syndrome. Of the subtypes of gastrointestinal adenomas, villous adenomas account for 5% of all adenomas [3]. Villous adenomas of the duodenum, however, are rare, often malignant neoplasms that account for roughly 1% of all duodenal tumors [4]. Despite their rarity, carcinomatous changes are reported in 30%-60% of these cases [4]. The size of the duodenal villous adenoma does not appear to correlate with the risk of malignancy, and treatment modalities remain controversial [4]. To our knowledge, we present the first biopsy-confirmed case of PLC originating from metastatic duodenal adenocarcinoma. This case highlights an exceptionally uncommon metastatic pathway and underscores the importance of recognizing rare gastrointestinal sources when evaluating diffuse pulmonary processes of unclear origin.

This case was presented as an abstract at the 2024 CHEST Annual Meeting on October 8, 2024.

## Case presentation

**Figure 1:**
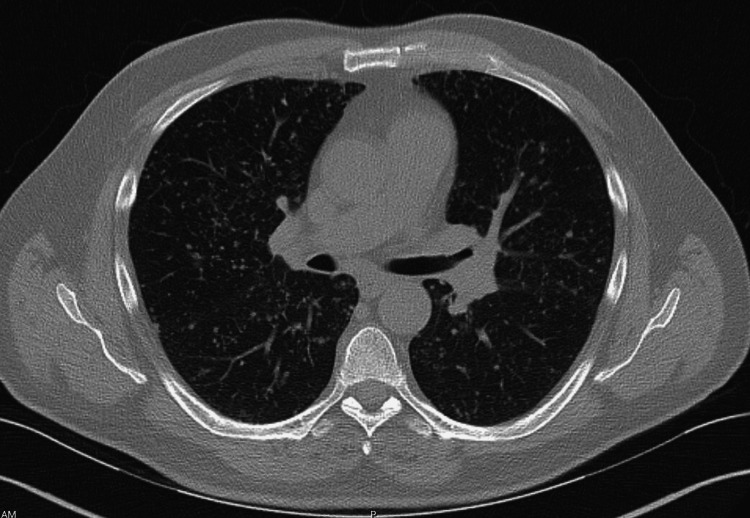
Innumerable subcentimeter, non-calcified pulmonary nodules bilaterally, with a miliary-like appearance and a random distribution pattern

A 56-year-old, non-smoker male with a history of hypercholesterolemia and non-insulin-dependent diabetes presented to his outpatient primary care physician's office with a complaint of intermittent dyspnea on exertion and a mildly productive cough with chest pain. These symptoms had been ongoing for four months after the patient developed a flu-like infection. The patient’s history subsequently revealed a 35-pound weight loss in that same time frame. He reported no fever, chills, hemoptysis, night sweats, or other constitutional symptoms. There was no family history of cancer, no recent travel, and no history of immunosuppressive medication use. He also had no history of significant environmental or work-related exposures. Initial laboratory evaluation revealed a normal complete blood count and comprehensive metabolic panel. A chest X-ray showed a diffuse, abnormal micronodular pattern throughout the bilateral lungs. A subsequent computed tomography (CT) scan revealed innumerable subcentimeter, non-calcified, diffusely scattered pulmonary nodules bilaterally, with an unusual miliary-like appearance, but no evidence of a primary neoplasm was visualized (Figure 1). A broad differential diagnosis was considered, including fungal, mycobacterial, and malignant etiologies. Fungal serology - including histoplasma, blastomycosis, and coccidioides antigens - and QuantiFERON TB testing were unrevealing, prompting the pursuit of a diagnostic bronchoscopy. Bronchoscopy with bronchoalveolar lavage was not diagnostic, with negative fungal and mycobacterial cultures.

**Figure 2:**
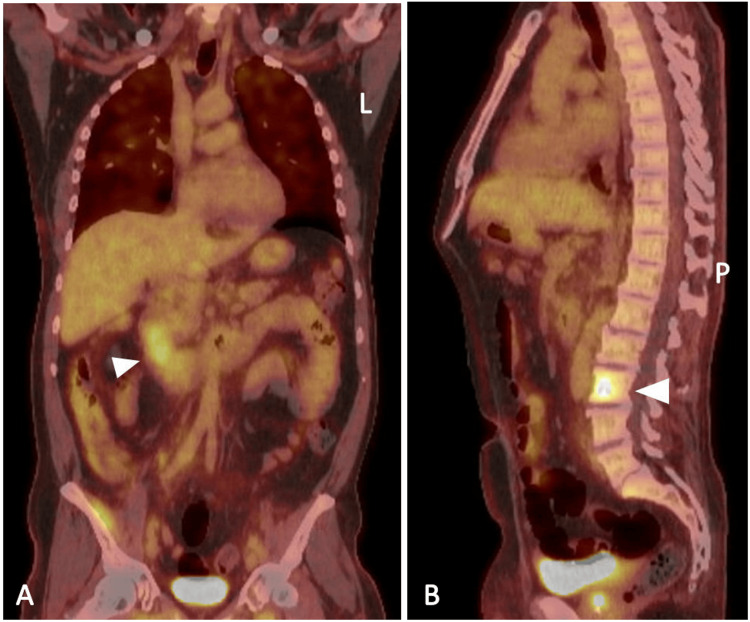
PET-CT scans (A) Coronal section showing focal uptake in the second/third portion of the duodenum (white arrowhead); (B) Sagittal section showing a hypermetabolic lesion in the L3 vertebral body (white arrowhead), consistent with osseous metastatic disease. PET/CT, Positron Emission Tomography/Computed Tomography

The patient was referred for video-assisted thoracoscopic surgery and underwent wedge resection of the right middle and right upper lobes. Pathology revealed invasive, moderately differentiated adenocarcinoma with multiple foci. Immunohistochemical stains performed on tumor cells were positive for cytokeratin 20 (CK20), MOC-31, special AT-rich sequence-binding protein 2 (SatB2) (focal), and caudal-type homeobox 2 (CDX2), but negative for paired box gene 8 (PAX8), cytokeratin 7 (CK7), thyroid transcription factor-1 (TTF-1), prostate-specific antigen (PSA), NK3 homeobox 1 (NKX3.1), and napsin A, with negative programmed death ligand 1 (PD-L1), suggestive of a gastrointestinal source as the etiology of the pulmonary nodules. Next-generation sequencing was obtained. Tumor mutation burden (TMB) was low, at three mutations per megabase of tumor DNA (mut/MB); for reference, low TMB is defined as 1-5 mut/MB. However, a mutation was identified in the KRAS (Kirsten rat sarcoma viral oncogene homolog) p.Gly12Val and ERBB3 p.Leu1177Ile regions. The differential diagnosis included metastasis from pancreaticobiliary or gastrointestinal tumors, or adenocarcinoma of other sites with enteric-type differentiation. A positron emission tomography (PET)/CT scan was obtained, which demonstrated low-level uptake within mediastinal lymph nodes, with a maximum standard uptake value (SUV) of 2.9, and heterogeneous, low-level uptake throughout the lung parenchyma, including an irregular 1.2 cm nodule with a maximum SUV of 2.5. This was accompanied by focal uptake at the second/third portion of the duodenum (Figure 2).

**Figure 3:**
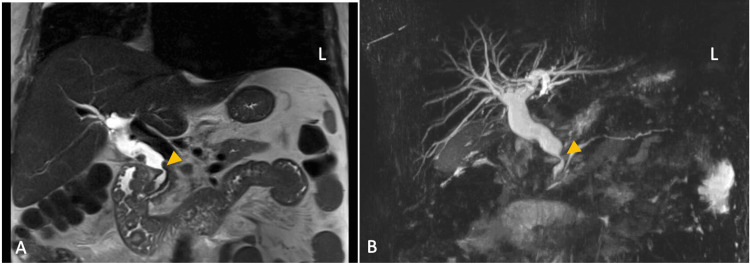
MRI abdomen (A) Coronal T2-weighted sequence showing a dilated CBD with abrupt cut-off (indicated by yellow arrowhead); (B) Coronal MIP demonstrating intrahepatic and extrahepatic biliary dilation with CBD stricture (indicated by yellow arrowhead). CBD, Common Bile Duct; MIP, Maximum Intensity Projection; MRI, Magnetic Resonance Imaging

**Figure 4:**
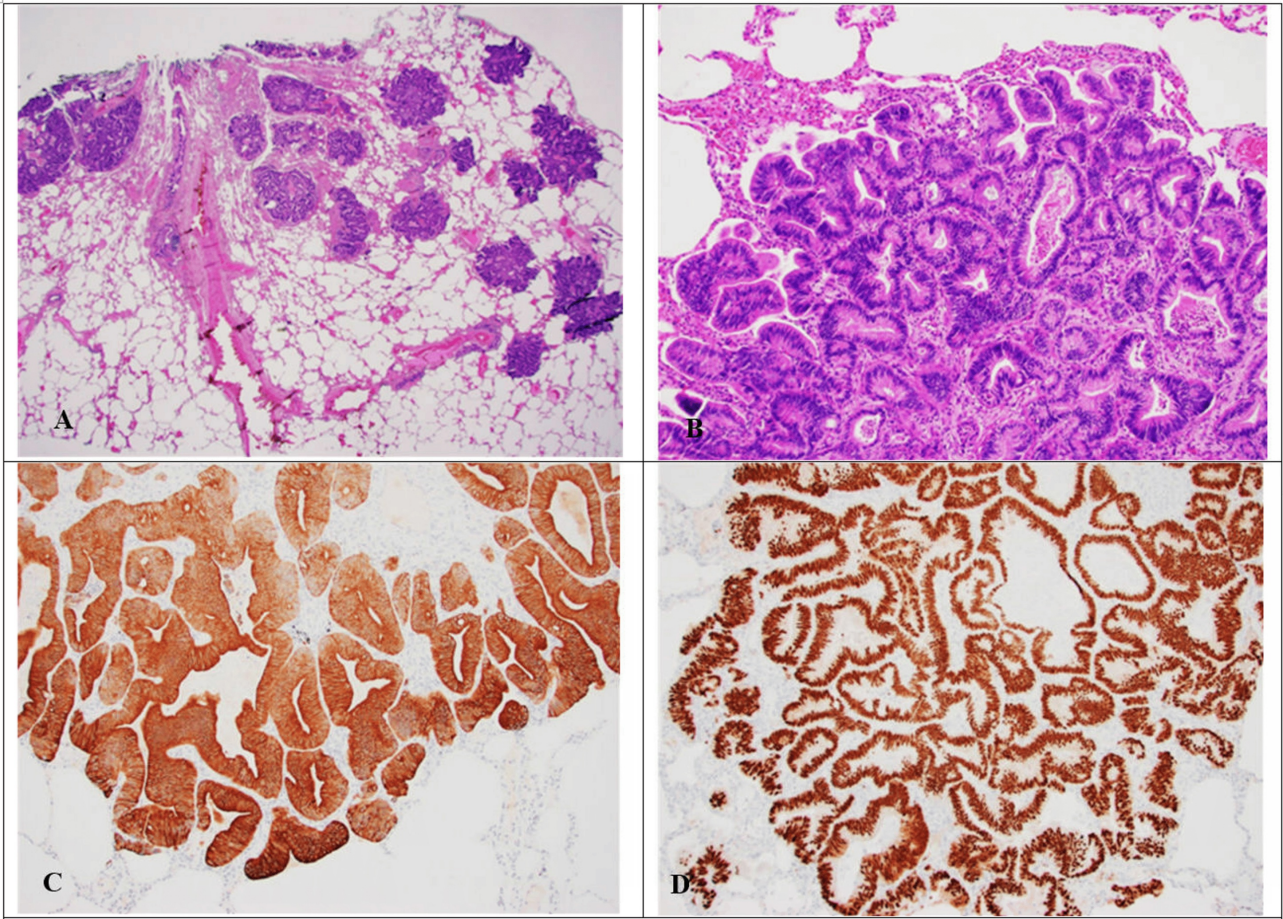
Histopathological images (A) Multiple foci of tumor metastasis (12.5× magnification, H&E); (B) Metastatic tumor cells forming glands with focal luminal necrosis (40× magnification, H&E); (C) Tumor cells staining diffusely and strongly positive for CK20 (100×, IHC); (D) Tumor cells staining diffusely and strongly positive for CDX2 (100×, IHC). H&E, Hematoxylin and Eosin; IHC, Immunohistochemistry

Given the radiographic findings and genetic markers, as evidenced by the KRAS gene mutation, our differential diagnosis narrowed to upper gastrointestinal involvement, including a pancreatic origin of malignancy. Magnetic resonance imaging (MRI) of the abdomen revealed an ill-defined, infiltrative mass arising from the pancreaticoduodenal groove, obstructing the distal common bile duct with marked upstream biliary duct dilation (Figure 3). However, due to the lack of pancreatic duct dilation, a duodenal origin of the adenocarcinoma was favored. Upper and lower endoscopies were performed, revealing a large fungating mass in the second portion of the duodenum, with histological confirmation of villous adenoma of the duodenum with low-grade dysplasia (Figure 4). There was no evidence of high-grade dysplasia or carcinoma from the duodenal biopsy, and no histopathologic abnormalities were noted in the large intestine. The patient's case was discussed at our facility's multidisciplinary tumor board, with a diagnostic consensus of metastatic primary duodenal adenocarcinoma with secondary PLC. The patient underwent 12 cycles of fluorouracil, leucovorin, oxaliplatin, irinotecan, and bevacizumab (FOLFOXIRI-Bev) chemotherapy.

**Figure 5:**
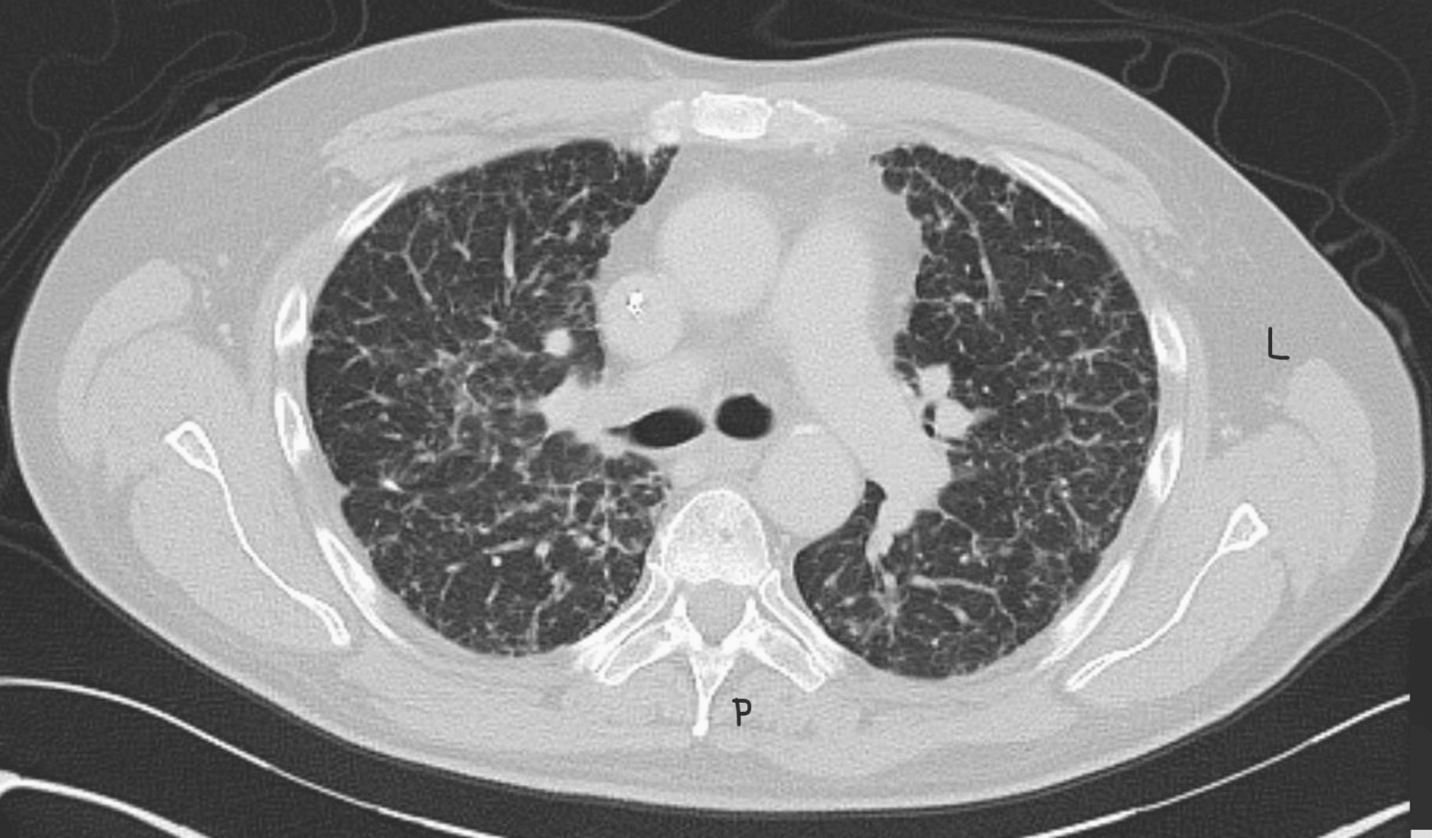
Worsening widespread pulmonary metastatic disease with extensive pulmonary nodules following completion of 12 cycles of chemotherapy

Following the completion of 12 cycles of FOLFOXIRI-Bev, a CT of the chest, abdomen, and pelvis revealed interval improvement of disease, with decreased lymphangitic carcinomatosis and decreased calcification of the soft tissue deposits in the chest wall. Therefore, the patient was initiated on capecitabine plus bevacizumab for maintenance therapy. Unfortunately, repeat CT imaging of the chest, abdomen, and pelvis six months later demonstrated a worsening miliary-like appearance of the lung parenchyma and new bony metastasis to the pelvic bones (Figure 5). The patient was reinitiated on FOLFOXIRI-Bev, in addition to monthly bisphosphonate infusions. He completed another 12 cycles of FOLFOXIRI-Bev, followed by a maintenance regimen of capecitabine with bevacizumab; unfortunately, further pulmonary and bony metastasis developed while on this regimen. His therapy was then modified to FOLFIRI-Bev, without 5-FU, as this approach was better tolerated and his declining functional status limited his ability to undergo intensive therapy. He received 13 cycles; however, a follow-up PET-CT scan revealed worsening tumor burden despite treatment. Given his worsening ECOG-PS (Eastern Cooperative Oncology Group Performance Status) score, a frank goals-of-care discussion took place, and a more comfort-centric approach was collectively agreed upon by the patient, his family, and the multidisciplinary care team. The patient expired 22 months after his initial diagnosis, in the comfort of his own home.

## Discussion

PLC is an uncommon but serious manifestation of metastatic malignancy, involving tumor cell infiltration into the pulmonary lymphatic system. It accounts for approximately 6%-8% of all pulmonary metastases and is most frequently associated with adenocarcinomas originating from the breast, stomach, pancreas, lung, and prostate [5]. Less common origins - including the colon, rectum, cervix, thyroid, liver, and melanoma - have also been reported [6]. The clinical presentation is often nonspecific, with symptoms such as dyspnea, nonproductive cough, and weight loss, which can overlap with more common respiratory diseases, making early recognition challenging [5].

Gastrointestinal primaries are an uncommon cause of PLC, with gastric cancer most frequently identified; duodenal adenocarcinoma remains exceedingly rare. In rare instances, oropharyngeal squamous cell carcinoma, squamous cell carcinoma of the cervix, and colon and lung cancers have been reported to present with lung metastasis in the form of PLC [7,8]. The expression of vascular endothelial growth factor-C by tumor cells induces lymphangiogenesis, which is thought to facilitate lymphatic metastasis to the lungs [9]. Given the rarity of gastrointestinal primaries causing PLC, imaging and histopathology become central to the diagnosis of both PLC and its primary.

Radiographically, chest imaging in PLC may show subtle early findings. Early disease can demonstrate interstitial lesions, linear and reticular shadows, and thickening of the interlobar fissures. As the disease advances, features such as irregular thickening of the bronchovascular bundles, interlobular septa, and the presence of small, beaded nodules along the interlobular septa and pleura become evident. The most significant CT finding in PLC is peribronchovascular thickening. High-resolution CT (HRCT) improves diagnostic accuracy, revealing thickening of the interlobular septa and bronchovascular interstitium, often with a “dot-in-box” appearance. Additional findings, such as subpleural nodules, interlobar fissure thickening, pleural effusions, and pleural carcinomatosis, can support the diagnosis. Nodules may present as miliary or reticulonodular patterns and can coalesce into a diffuse interstitial pattern [10]. In this case, numerous subcentimeter, non-calcified nodules were diffusely scattered throughout both lungs in a nonspecific distribution. PET-CT provides both anatomical and functional information, revealing areas of increased uptake - particularly in the pulmonary lymphatic system - which suggests active tumor spread. These imaging findings can assist in staging and guide therapeutic decisions. PET-CT is especially valuable for identifying occult primary malignancies and assessing disease extent, with 86% sensitivity and 100% specificity in detecting PLC. As demonstrated, heterogeneous low-level uptake in the lung parenchyma, along with pulmonary nodules, assisted in the diagnosis [10-13]. The radiographic appearance of PLC can mimic other interstitial lung diseases, such as pulmonary edema, sarcoidosis, or silicosis [14]. Understanding these radiographic patterns is essential not only for early detection but also for appreciating the underlying metastatic pathways that facilitate pulmonary lymphangitic spread. 

Several theories have been proposed to explain the metastatic mechanisms of PLC. The metastatic process may involve cancerous invasion of mediastinal and pulmonary hilar lymph nodes, obstructing drainage and leading to retrograde lymphatic spread. Alternatively, hematogenous spread may occur via vascular endothelium damage, ultimately reaching the perivascular lymphatics and allowing direct entry of malignant cells. Another postulated mechanism includes a trans-diaphragmatic route, where tumor cells trapped in the lymphatic vessels cause local obstruction and inflammation, thickening the bronchovascular and alveolar septa due to tissue edema. The underlying mechanisms driving this malignancy are not yet fully elucidated [1].

Molecular alterations play a central role in the pathogenesis of primary duodenal adenocarcinoma and can influence its metastatic behavior. Gain-of-function mutations in the β-catenin gene that result in nuclear accumulation of β-catenin often drive primary duodenal adenocarcinoma. Additional mutations in KRAS, tumor protein p53 (TP53), and deleted in colorectal carcinoma (DCC) further contribute to malignant transformation [15]. Among these, KRAS mutations - present in approximately one-third of duodenal adenocarcinomas - are associated with poor tumor differentiation, higher stage at diagnosis, and increased risk of distant relapse, though their independent prognostic significance is limited after adjusting for nodal status. Importantly, KRAS mutations predict resistance to anti-EGFR (epidermal growth factor receptor) therapies (e.g., cetuximab and panitumumab), guiding therapeutic decision-making, and RAS testing is recommended in small bowel adenocarcinomas by extrapolation from colorectal cancer guidelines [16]. ERBB (HER2) gene alterations are found in about 12% of small bowel adenocarcinomas and more commonly in duodenal primaries. This alteration may identify patients who could benefit from anti-HER2 therapy, although prospective data are lacking [17]. In our patient, while the duodenal biopsy lacked high-risk features, PET-CT imaging, lung wedge resection histology, and immunohistochemistry (including CK20, CDX2, SatB2, and MOC-31) identified the duodenum as the primary tumor site. These molecular and immunohistochemical markers were critical in confirming the tumor’s origin and have direct clinical relevance for guiding targeted therapy selection.

While molecular profiling helps develop potential targeted therapies, accurate determination of the primary site in metastatic pulmonary lesions relies heavily on immunohistochemical profiling. CK20 is strongly associated with colorectal adenocarcinoma, with one study reporting up to 95% positivity in pulmonary metastases of colorectal origin [18]. MOC-31, a monoclonal antibody targeting epithelial cell adhesion molecule (EpCAM), aids in identifying adenocarcinomas of epithelial origin [19]. SatB2 and CDX2 are markers of intestinal differentiation - particularly in colorectal cancer - and their combined expression enhances diagnostic accuracy [20]. In this scenario, the presence of CK20, CDX2, SatB2, and MOC-31 expression in the lung lesions raised a strong suspicion for a gastrointestinal origin, later confirmed by a duodenal biopsy showing a villous adenoma.

While imaging findings raise suspicion, a definitive diagnosis of PLC requires a lung biopsy; however, the procedure is frequently deferred due to its invasive nature and the poor respiratory status of many patients with this condition. Histopathological examination confirms the diagnosis, showing interstitial thickening, lymphatic infiltration, and occasionally granulomatous inflammation [1]. Conventional pulmonary arteriography and endobronchial ultrasound (EBUS) aid in the diagnostic process of PLC or can differentiate it from pulmonary tumor embolism, which can mimic PLC. Immunohistochemistry distinguishes PLC from other mimicking conditions. Most diagnoses are made postmortem, highlighting the diagnostic difficulty and the aggressive nature of the disease [1].

Management of PLC is challenging, as it typically reflects advanced metastatic disease. Treatment is largely palliative, aiming to relieve symptoms and improve quality of life, since curative options are generally not feasible. Chemotherapy may be used depending on the responsiveness of the primary malignancy, though outcomes are variable. Supportive measures, such as oxygen therapy and pain control, are essential, and multidisciplinary care is critical to address respiratory and systemic complications. Surgical resection is rarely feasible and is typically reserved only for isolated disease. Prognosis is generally poor, with median survival ranging from a few months to a year. Early detection may improve outcomes. Notably, although diagnosed late, our patient survived for 22 months after the initial diagnosis.

## Conclusions

PLC is typically identified in patients with an established history of malignancy; however, in rare instances, it can serve as the initial manifestation of an occult cancer. This case report presents the first biopsy-confirmed instance of PLC originating from a primary duodenal villous adenocarcinoma. Duodenal adenocarcinomas account for approximately 50%-60% of all duodenal malignancies, while the villous subtype constitutes only 10%-20% of these. The diagnostic process in this instance was particularly challenging, given the nonspecific pulmonary symptoms, absence of a known primary malignancy, and indeterminate initial imaging studies. PET-CT imaging, histopathological analysis, immunohistochemical profiling, and multidisciplinary collaboration were crucial in identifying the duodenum as the primary tumor source. This case adds to the limited body of literature by documenting an atypical presentation of PLC and emphasizes the diagnostic utility of combining imaging with molecular and pathological tools in evaluating diffuse pulmonary processes of unclear origin. Reporting such rare associations is vital for enhancing clinical recognition, informing diagnostic strategies, and ultimately improving outcomes in patients with atypical metastatic disease presentations.

## References

[1] Ajith Kumar AK, Mantri SN (2025). Lymphangitic carcinomatosis. StatPearls [Internet].

[2] Kim GE, Siddiqui UD (2023). Endoscopic resection techniques for duodenal and ampullary adenomas. VideoGIE.

[3] Myers DJ, Arora K (2025). Villous adenoma. StatPearls [Internet].

[4] Verma A, Kumar S (2013). Villous adenoma of duodenum: a rare case presentation with review of literature. Indian J Surg Oncol.

[5] Klimek M (2019). Pulmonary lymphangitis carcinomatosis: systematic review and meta-analysis of case reports, 1970-2018. Postgrad Med.

[6] Zhuang L, Liu X, Hu C, Zhang L, Jiang G, Wu J, Zheng S (2014). Pulmonary lymphangitic carcinomatosis in liver carcinoma: a rare case report and literature review. World J Surg Oncol.

[7] Kanthan R, Senger JL, Diudea D (2010). Pulmonary lymphangitic carcinomatosis from squamous cell carcinoma of the cervix. World J Surg Oncol.

[8] Otsubo K, Kubo N, Nakashima N, Izumi M, Nakamori M, Koto H (2011). A juvenile case of pulmonary lymphangitic carcinomatosis caused by sigmoid colon cancer with a component of micropapillary carcinoma. Intern Med.

[9] Das S, Ladell DS, Podgrabinska S, Ponomarev V, Nagi C, Fallon JT, Skobe M (2010). Vascular endothelial growth factor-C induces lymphangitic carcinomatosis, an extremely aggressive form of lung metastases. Cancer Res.

[10] Wu JW, Chiles C (1999). Lymphangitic carcinomatosis from prostate carcinoma. J Comput Assist Tomogr.

[11] Prakash P, Kalra MK, Sharma A, Shepard JA, Digumarthy SR (2010). FDG PET/CT in assessment of pulmonary lymphangitic carcinomatosis. AJR Am J Roentgenol.

[12] Grabill N, Louis M, Cawthon M, Gherasim C, Walker T (2024). Duodenal adenocarcinoma at stage IV: a critical look at diagnostic pathways and treatment modalities. Radiol Case Rep.

[13] Jreige M, Dunet V, Letovanec I, Prior JO, Meuli RA, Beigelman-Aubry C, Schaefer N (2020). Pulmonary lymphangitic carcinomatosis: diagnostic performance of high-resolution CT and 8F-FDG PET/CT in correlation with clinical pathologic outcome. J Nucl Med.

[14] Honda O, Johkoh T, Ichikado K (1999). Comparison of high resolution CT findings of sarcoidosis, lymphoma, and lymphangitic carcinoma: is there any difference of involved interstitium?. J Comput Assist Tomogr.

[15] Puccini A, Battaglin F, Lenz HJ (2018). Management of advanced small bowel cancer. Curr Treat Options Oncol.

[16] Sepulveda AR, Hamilton SR, Allegra CJ (2017). Molecular biomarkers for the evaluation of colorectal cancer: guideline from the American Society for Clinical Pathology, College of American Pathologists, Association for Molecular Pathology, and the American Society of Clinical Oncology. J Clin Oncol.

[17] Laforest A, Aparicio T, Zaanan A (2014). ERBB2 gene as a potential therapeutic target in small bowel adenocarcinoma. Eur J Cancer.

[18] Al-Maghrabi J, Emam E, Gomaa W (2018). Immunohistochemical staining of cytokeratin 20 and cytokeratin 7 in colorectal carcinomas: four different immunostaining profiles. Saudi J Gastroenterol.

[19] Hecht JL, Pinkus JL, Pinkus GS (2006). Monoclonal antibody MOC-31 reactivity as a marker for adenocarcinoma in cytologic preparations. Cancer.

[20] Shakweer MM, Meckawy GR (2025). Special AT-rich sequence-binding protein 2 (SATB2) expression in colorectal carcinoma; a possible future perspective for targeted therapy. Immunopathologia Persa.

